# Strategies to Functionalize the Anionic Biopolymer Na-Alginate without Restricting Its Polyelectrolyte Properties

**DOI:** 10.3390/polym12040919

**Published:** 2020-04-15

**Authors:** Luca Szabó, Sandrine Gerber-Lemaire, Christine Wandrey

**Affiliations:** Group for Functionalized Biomaterials, Institute of Chemical Sciences and Engineering, Ecole Polytechnique Fédérale de Lausanne, EPFL SB ISIC SCI-SB-SG, Station 6, CH-1015 Lausanne, Switzerland; luca.szabo@epfl.ch (L.S.); christine.wandrey@epfl.ch (C.W.)

**Keywords:** alginate-based copolymers, covalent cross-linking, hybrid hydrogels, ionically cross-linked hydrogels, polymer functionalization, sodium alginate

## Abstract

The natural anionic polyelectrolyte alginate and its derivatives are of particular interest for pharmaceutical and biomedical applications. Most interesting for such applications are alginate hydrogels, which can be processed into various shapes, self-standing or at surfaces. Increasing efforts are underway to functionalize the alginate macromolecules prior to hydrogel formation in order to overcome the shortcomings of purely ionically cross-linked alginate hydrogels that are hindering the progress of several sophisticated biomedical applications. Particularly promising are derivatives of alginate, which allow simultaneous ionic and covalent cross-linking to improve the physical properties and add biological activity to the hydrogel. This review will report recent progress in alginate modification and functionalization with special focus on synthesis procedures, which completely conserve the ionic functionality of the carboxyl groups along the backbone. Recent advances in analytical techniques and instrumentation supported the goal-directed modification and functionalization.

## 1. Introduction

Alginates (alg) are natural anionic polyelectrolytes, which are quite abundant in nature [1]. Commercial alg are generally produced by extraction from marine brown algae, though some bacteria are able to synthesize alg as well [2]. Microbial biosynthesis is possible, yielding alg over a very wide range of tailored composition [3,4]. The global production of alg and alg derivatives increased from about 48.73 × 10^6^ t in 2010 to 58.27 × 10^6^ t in 2015 with an average annual growth rate predicted as more than 3.65% per year.

Alg, including alginic acid and its salts, are traditionally used as stabilizers, thickeners, emulsifiers, as well as hydration and gelling agents in the food, beverage, cosmetics, paper, textile printing, and pharmaceutical industries. Alg have also found several applications in biomedical science and engineering due to a number of properties that are favorable for the biomedical field. In particular, alg hydrogels are promising in wound healing, drug delivery, and regenerative medicine applications. Depending on the intended use, different requirements concerning the purity, chemical composition, and physical characteristics are made. Only certified medically grade alg, which complies with the limits of endotoxin and other toxic impurities, is allowed for medical applications.

Due to the frequently unfavorable mechanical stability, integrity, deformability, and permeability of purely ionically cross-linked alg hydrogels, which hinder the progress of sophisticated biomedical applications, increasing efforts are underway to functionalize the alg macromolecules prior to hydrogel formation in order to overcome these shortcomings. Special attention is given to derivatives of alg, which allow simultaneous ionic and covalent cross-linking to improve the physical properties of the hydrogel and likewise add biological activity adapted to the intended application.

In terms of regulatory aspects for modified alg intended for biomedical applications, not only appropriate purity is required but also SOPs (Standard Operation Protocols) and GMP (Good Manufacturing Practice) certification of the modification procedures may become necessary before translation to the clinics.

This review will report recent progress in alg modification and functionalization with special focus on synthesis procedures, which completely conserves the ionic functionality of the carboxyl groups along the backbone, or at least to such an extent that fast ionic gelation can still occur. Following a brief presentation of alg as the raw material for functionalization, the review will mainly address the methodologies for modification of the hydroxyl groups of the alg backbone, including direct derivatization or partial oxidation of alg vicinal diols followed by further conjugation with nucleophilic derivatives.

## 2. Alginate: Composition and Physicochemical Properties

### 2.1. Chemical Description of Alginate

**Figure 1:**
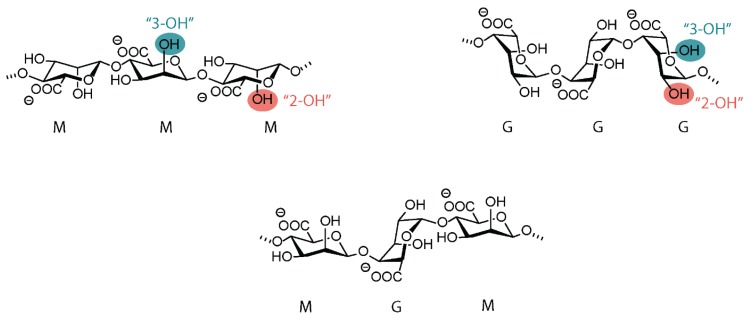
Chemical structure of alginate. C2 and C3 hydroxyl groups, which are amenable to chemical functionalization, are highlighted.

The term alg covers a family of polysaccharide structures consisting of linear copolymers of (1→4) linked β-D-mannuronic acid (M) and α-L-guluronic acid (G) units [1,5,6,7]. The linear backbones are composed of regions of G- and M-blocks interspaced with regions of mixed sequences, MG-blocks [8]. Figure 1 shows exemplary sections of alg backbones. Depending on the type of the seaweed, the quality of the extract, the isolation process, and the biotechnological procedure, the proportion and sequential arrangement of the two structural units can strongly vary. M and G blocks of various lengths can constitute the polymer chain. The weight average molar mass of commercial Na-alg varies from 4 × 10^4^ to 5 × 10^5^ g/mol. However, some alg with molar masses higher than 10^6^ g/mol have also been isolated. For the same average molar mass, the molar mass distribution can be quite different.

### 2.2. Physicochemical and Polyelectrolyte Characteristics of Alginate

The composition, internal structure, and molar mass of the alg backbone govern the functional properties [9]. The solubility of alg in water depends on the pH value and the type of the counterions of the carboxyl groups. At pH < 3, both the M- and G-structures will precipitate as alginic acid. However, alternating M- and G-structures precipitate at lower pH values compared to the alg containing more homogeneous block structures. Neutralization of the alginic acid occurs at pH > 4, where it is converted into its corresponding salt [10]. Na-alg is an example of a water-soluble alg.

Na-alg behaves in aqueous solution as a typical linear homogeneously charged polyelectrolyte. The chain conformation and consequently the solution viscosity strongly depend on the ionic strength, i.e., on both the alg concentration and the simultaneous presence of low molar mass salt. The intramolecular electrostatic repulsion between the neighboring negatively charged carboxyl groups of each monomer unit forces alg molecules into an extended random coil conformation in aqueous solution [11]. This results in highly viscous solutions even at relatively low alg concentration. The dynamic viscosity increases exponentially with the molar mass, while the intrinsic flexibility of the alg chains in solution increases in the order GG < MM < MG [12]. On the other hand, the selectivity for cation-binding and gel-forming properties strongly depends on the composition and sequence. Divalent cations preferably bind to the G-blocks. The ability to form ionotropic gels is based on this selective binding of cations. Mixing solutions of Na-alg with solutions of cationic polyelectrolytes yields precipitates of polyelectrolyte complexes.

The rheological behavior of Na-alg solutions is important for a number of technologies and depends on both the concentration and the shear rate. Higher concentrated solutions exhibit pseudoplastic flow even at low shear rates. At lower concentrations, the Newtonian behavior is observed at low shear rate, whereas the solutions become pseudoplastic at a higher shear rate [1].

Na-alg as a dry powder and, in particular as a solution, is subject to degradation, leading to the rapid decrease of the chain length. Many microorganisms digest alg. In a deep freezer, Na-alg may be kept for several years without significant degradation. Purified and sterilized samples better resist degradation.

## 3. Modification with Partial Conservation of the Carboxyl Groups

**Figure 2:**
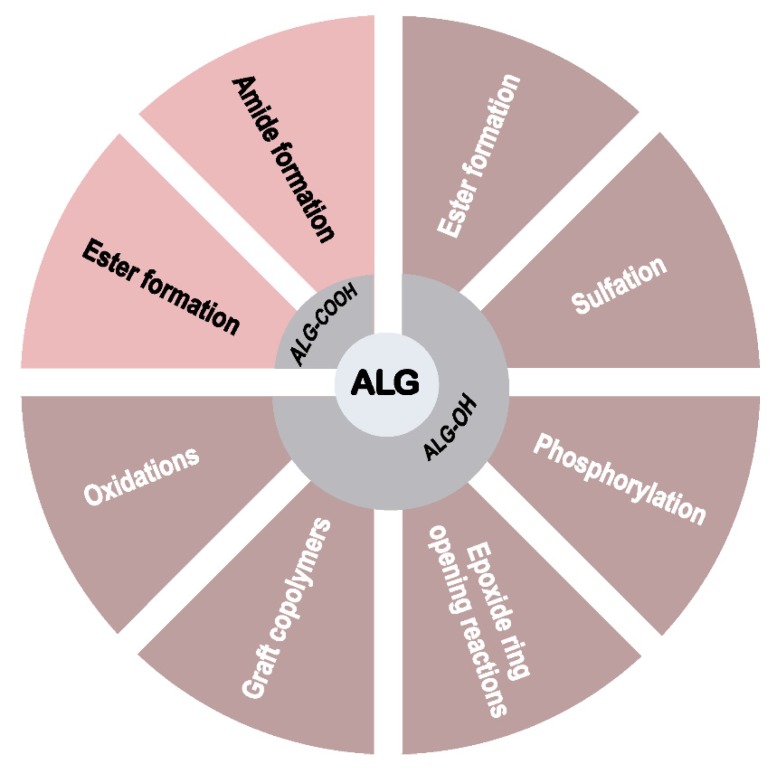
Chemical transformations for the functionalization of alginates.

The backbone of Na-alg presents a series of carboxyl and hydroxyl groups amenable to chemical functionalization (Figure 2) [13]. Before moving to the primary focus of the review, the modification of Na-alg with complete conservation of the carboxyl groups, progress concerning only partial conservation is briefly summarized.

While Na-alg is known to undergo fast gelation in the presence of divalent cations such as Ca^2+^ and Ba^2+^ [14], the synthesis of alg derivatives is used to modulate the properties of the resulting hydrogels. Not only the physical characteristics (mechanical resistance, elasticity, stability under physiological conditions) of alg hydrogels can be modified through chemical functionalization of the starting polymers, but also their in vitro and in vivo compatibility is largely influenced by conjugation to other polymers or small molecular scaffolds.

Due to the large variety of mild activating agents to increase the reactivity of carboxyl groups, one of the most straightforward chemical modifications of Na-alg relates to the partial conversion of its carboxylates into ester and amide derivatives. In particular, the combination of 1-ethyl-3-(3-dimethylaminoprolyl) carbodiimide (EDC) and *N*-hydroxysuccinimide (NHS) was largely reported for the formation of alg-derived amides with variable degrees of grafting. Using this methodology, Na-alg was functionalized with dopamine at 6.6% of the carboxyl groups [15]. The resulting polymer was applied to a multilayer coating of bioprosthetic heart valves, causing efficient anti-calcification protective effects due to dynamic calcium–sodium ionic exchange. Using a similar functionalization pathway, tyramine-conjugated planar and spherical alg hydrogels were produced to overcome the bioinertness of alg scaffolds [16]. The functionalized scaffolds presented elastic moduli similar to human tissues, enhanced the adsorption of proteins in cell culture media, and allowed the stable adhesion of human mesenchymal stem cells and human pluripotent stem cell-derived cardiomyocytes. Dopamine-modified alg was also used for the formation of covalently cross-linked hydrogels under oxidizing conditions (sodium periodate) [17]. While this cross-linking strategy did not induce cytotoxicity on murine pancreatic islets, it demonstrated a marked detrimental effect on stimulated insulin secretion in comparison with Na-alg encapsulation material. 

In view of the potential of alg hydrogels for the microencapsulation and subsequent transplantation of endocrine cells, modification of the alg carboxylic groups was investigated to improve both the mechanical properties and biocompatibility of the resulting microspheres (MS). The conjugation, through amide formation, of Na-alg with perfluorinated (PF) alkyl groups using a short poly(ethylene glycol) (PEG) spacer allowed the production of ionic PF-alg MS with better swelling behavior than pure Ba-alg MS. With only 3.3% of PF chain content, PF-alg MS enhanced the viability of mouse insulinoma MIN6 cells, while presenting similar permeability profiles as Ba-alg MS [18].

Cross-linked alg hydrogels were further obtained using functionalized cross-reactive alg derivatives produced by amide formation on the alg carboxylate groups. Copper-catalyzed azide to alkyne [3+2]-cycloaddition—namely click-reaction—increased the stability of ionic alg hydrogels in aqueous ethylenediaminetetraacetic acid (EDTA) solution due to additional triazole cross-linking [19]. The resulting MS displayed higher permeability toward low molar mass components and lower gel swelling than simply ionically cross-linked alg hydrogels. Alternatively, the amide coupling of Na-alg with 3-aminophenylboronic acid afforded 25% of grafting, as determined by ^1^H-NMR spectroscopy [20]. The resulting boronic acid-containing alg derivatives underwent chemical cross-linking with the vicinal diols present on the alg pyranose rings to produce injectable multi-stimuli responsive and self-healing hydrogels [20]. Other bio-orthogonal reactions, such as Diels–Alder cycloaddition and Staudinger ligation, were reported for the covalent cross-linking of furan-containing and azido-containing alg derivatives, respectively [21,22]. The partial conversion of alg carboxylates into amide and ester analogues was exploited for the formation of combinatorial hydrogel libraries aiming at the identification of chemical motifs that are able to mitigate the foreign body response after transplantation [23]. A triazole-containing alg derivative used to transplant human glucose-responsive mature beta cells in immune competent diabetic mice demonstrated excellent performance [24]. Finally, amide formation on the carboxylate groups of alg was applied to produce alg–peptide bioconjugates for tissue engineering [25] and silica–alg core–shell nanoparticles for cisplatin-based anticancer prodrugs delivery [26].

## 4. Modification with Complete Conservation of the Alginate Carboxyl Groups

### 4.1. Ester Formation

#### 4.1.1. Acetylation

One of the earliest chemical modifications performed on alginic acid was acetylation of the alg hydroxyl groups. This modification did not provide alg derivatives with significantly new characteristics nor was it for any specific applications. However, it was particularly important in the understanding of some alg features and reactivity at the early stages.

**Figure 3:**
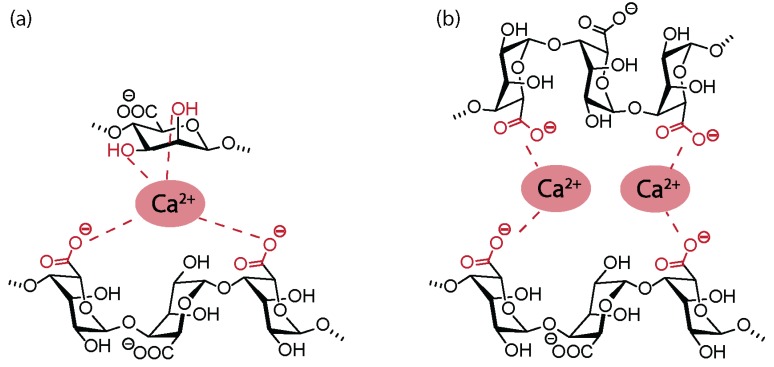
(**a**) Early hypothetical coordination model for Ca^2+^ alginate gels. (**b**) Egg-box model for the interaction of alginate with divalent cations.

In 1946, Wassermann reported the successful acetylation of alginic acid, Na-alg, and Ca-alg in the presence of ketene, reaching approximatively one acetate group per saccharide unit, i.e., a degree of grafting (DG) = 1 [27,28]. The same year, it was shown that swelling alginic acid in water, followed by an almost complete exchange to glacial acetic acid, could significantly reduce the extent of chain degradation [29]. The same reaction could not be performed on dry alginic acid, as the hydrogen bonds between neighboring molecules were found to be too strong, and the structure was found to be too compact for the acetylation reaction to happen. Under similar conditions, Schweiger reported the acetylation of alginic acid with a maximal DG of 1.85 [30] and pointed toward the increase of the reaction rate following the acetylation of one hydroxyl group due to the disruption of the strong hydrogen bonds between vicinal hydroxyl groups. Acetylation reactions were also used to investigate the insolubility and precipitation of alg salts [31]. While alg were expected to gelify and eventually precipitate in the presence of increasing concentration of di- or trivalent cations, diacetylated alg derivatives did not undergo gelification in the presence of Ca^2+^ ions. Surprisingly, even with one free hydroxyl group per saccharide unit (DG = 1), precipitation could not be observed in the presence of multivalent cations. Based on these results, Schweiger et al. proposed that the coordination of Ca^2+^ ions in alg-derived gels involved not only the carboxylate moieties but also two vicinal hydroxyl groups (Figure 3). Later investigations led to the revision of this coordination structure in favor of the egg-box model [32].

Acetylation reactions also served to establish the role of O-acetylated groups in the protective effect of alg in bacteria [33], preventing enzymatic degradation of the polysaccharide backbone [34]. Investigation of the effect of the degree of acetylation on the macromolecular properties of alg pointed toward the increased swelling ratio of acetylated Ca-alg gels [35]. Viscosity measurements indicated that a low degree of acetylation results in more stiffness and the expansion of the alg chain molecules, while a highly acetylated alg causes a more flexible polymer chain [35].

More recently, the acetylation of tetrabutylammonium (TBA)-alg in organic solvents led to a maximum DG of 1, raising the question of the regioselectivity of the reaction (C2–OH versus C3–OH) or the different reactivity of M and G units [36]. It was hypothesized that 1,3-diaxial interactions of C2–OH groups with C4 protons in M residues and of C3–OH groups with C5 protons in G residues were responsible for the acetylation of a single hydroxyl group per saccharide unit.

#### 4.1.2. Other Esterification Methods

**Figure 4:**
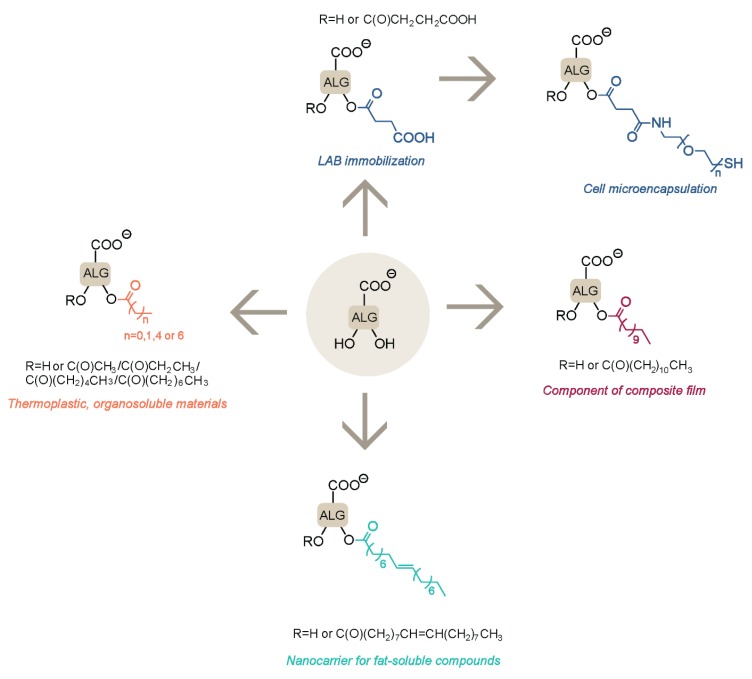
Alginate ester derivatives and their applications.

In order to maintain the full capacity of alg carboxylate moieties for ionotropic interactions, esterification of the hydroxyl groups was often proposed to modify the properties of alg hydrogels without reducing alg gelling properties (Figure 4). Alg beads were reported for the immobilization of lactic acid bacteria (LAB), which are essential for a healthy intestinal flora but are very sensitive to the acidic pH of gastric fluid (pH < 2). In comparison with Ca-alg beads, succinylated hydrogels improved the viability of LAB in acidic media by assisting to the neutralization of acidic pH due to an increased number of carboxylate functionalities on the polysaccharide backbone [37]. Similarly, the palmitoylation of Na-alg resulted in an increased resistance of immobilized LAB due to the higher hydrophobic character of the resulting beads, thus restricting the amount of gastric fluid reaching the inner core of the hydrogel. 

Alg esterification by succinic anhydride was also used to introduce an additional carboxylic moiety on the alg backbone, allowing further functionalization through amide formation. This strategy was reported for the conjugation of Na-alg with heterobifunctional PEG derivatives equipped with end-thiol moieties [38]. The resulting PEG-grafted alg led to the formation of spherical hydrogels assembled by dual ionotropic interactions with Ca^2+^ ions and covalent disulfide bridges, without the need for an additional external chemical cross-linker. These hydrogels showed improved mechanical resistance and shape recovery performance in comparison with pure Ca-alg beads, as well as favorable properties for the microencapsulation of human foreskin fibroblasts. The esterification of alg was performed as well in the absence of organic solvents, using formic acid to disrupt the network of hydrogen bonds, thus facilitating further reaction of the hydroxyl groups with fatty acid chlorides [39]. The resulting hydrophobic alg derivatives were used as nanocarriers for the controlled release of Vitamin D3.

Several alginic acid ester derivatives with different acyl chain lengths were prepared as new thermoplastic, organosoluble materials [40]. The use of perchloric acid catalyst resulted in the chemical modification of all alg hydroxyl groups, but it was associated with significant chain degradation as evaluated by gel permeation chromatograpy (GPC) measurements. Finally, the derivatization of Na-alg with lauroyl chloride was developed for incorporation into psyllium husk–gel composite films [41], conferring improved mechanical strength and antibacterial activity to the materials.

### 4.2. Sulfation

**Figure 5:**
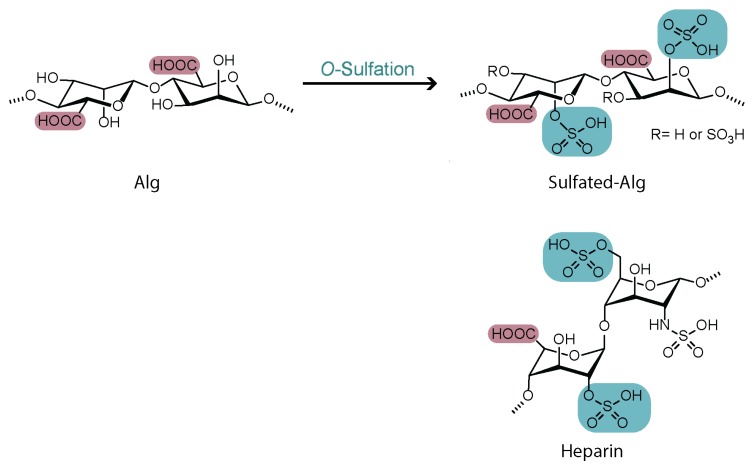
Structural similarities between heparin and sulfated alginate derivatives.

Most *O*-sulfation reactions on alg are aiming to provide the polysaccharide with features similar to those of heparin. Heparin, a naturally occurring glycosaminoglycan, is a widely used anticoagulant that is composed of linearly repeated sulfated disaccharide units of hexuronic acid and hexosamine, displaying both carboxylates and sulfated functional groups [42]. Being the only naturally occurring polysaccharide containing carboxylic acid moieties in each of its uronic acid units, alg appeared as an excellent candidate to develop heparin analogues by sulfation of the hydroxyl functionalities (Figure 5).

Many conditions were reported for the synthesis of sulfated alg derivatives (Table 1). Due to its low cost and ease of use, chlorosulfonic acid is the most common reported sulfation reagent, allowing to achieve various degrees of functionalization with good reproducibility [43,44,45,46,47,48]. In order to prevent degradation of the polysaccharide backbone, N(SO_3_Na)_3_ was reported as a mild, non-toxic, and low-cost reagent for the sulfation of Na-alg in aqueous medium [49]. The use of coupling reagents such as dicyclohexylcarbodiimide (DCC) activated by sulfuric acid was also proposed for the modification of TBA-alg [50].

The degree of sulfation was probed with several analytical techniques, including FT-IR, elemental analysis, and NMR spectroscopy. While the maximum reported DG was around 1, the optimal sulfation degrees for applications in ionotropic gel formation were reported to be between 0.64 and 0.8 [51]. In general, no significant regioselectivity was observed between the C2–OH and C3–OH substitution; however, a slight preference for the sulfation of the C2–hydroxyl groups was reported for both M and G units [45,47].

In addition to the exploitation of the heparin-like properties, sulfated alg derivatives were also successfully applied to the entrapment of cell-adhesion molecules, growth factors, and chemokines. The controlled and sustained delivery of the basic fibroblast growth factor (bFGF) was achieved with MS produced from a mixture of Na-alg and sulfated alg [50]. Similarly, the encapsulation of chondrocytes in sulfated-alg 3D matrices supported high cell viability and preserved their phenotype [46,51]. Sulfated alg derivatives were also shown to have increased anti-viral activity compared to unmodified polymers. The higher the degree of sulfation, the higher the anti-viral potency was found against herpes virus type 1 (HSV-1) [52]. 

A clinically relevant sulfated derivative is the propylene glycol alg sodium sulfate (PSS), which was the first oral heparinoid drug to be approved in China in 1987, and it has been used since then in China both orally and intravenously for the treatment of cardiovascular diseases [53]. PSS derivatives with various sulfur content, molar mass, and G:M ratios were intensively investigated for their P-, L-, and E-selectin mediated binding to tumor cells [48].

### 4.3. Phosphorylation

To the best of our knowledge, there are only two relevant examples in the literature for the phosphorylation of the alg hydroxyl groups. One study discusses the *O*-phosphorylation of Na-alg and the effect of the resulting phosphorylated derivatives on the induction of hydroxyapatite (HAP) nucleation and growth [54]. In the presence of phosphoric acid and urea, the phosphorylation of Na-alg was accompanied by significant chain degradation, leading to a reduction of the average molar mass by a factor of 2 to 4. While the functionalization of M residues occurred preferentially at the C3–OH groups, the regioselectivity on G residues could not be elucidated from 1D- and 2D-NMR analyses. Due to substantial chain degradation and possible conformational changes upon modification, phosphorylated alg derivatives had to be mixed with native Na-alg to achieve hydrogel formation by extrusion into a Ca^2+^ containing solution.

Secondly, Na-alg *O*-phosphorylation was achieved by using a mixture of H_3_PO_4_/P_2_O_5_/Et_3_PO_4_/hexanol. The resulting phosphorylated alg derivatives were subsequently submitted to cation exchange by Ca(OAc)_2_ and mixed with Na-alg, in different ratios to produce hydrogels resulting from an internal gelation process [55]. The suitability of these materials for cell immobilization was demonstrated on MC3T3 cells.

### 4.4. Epoxide Ring-Opening Reactions

Modulation of the hydrophilic character of alg was achieved by covalent conjugation to long linear alkyl chains through epoxide ring-opening reactions [56]. The condensation of Na-alg to dodecyl glycidyl ether, under basic conditions, resulted in amphiphilic alg derivatives that are able to self-assemble into micelles in aqueous solutions. The potential of these polymers to produce efficient drug carriers was evaluated on the highly hydrophobic drugs Clofazimine and Amphotericin B, showing a moderate increase of their apparent solubility, in comparison with cyclodextrin- or liposomic-based formulations [57]. In a subsequent report, an improved synthetic route for the preparation of amphiphilic alg derivatives was presented, by addition of sodium dodecyl sulfate (SDS) and orthogonal optimization of the reaction conditions to achieve higher reaction yields [58]. 

**Figure 6:**
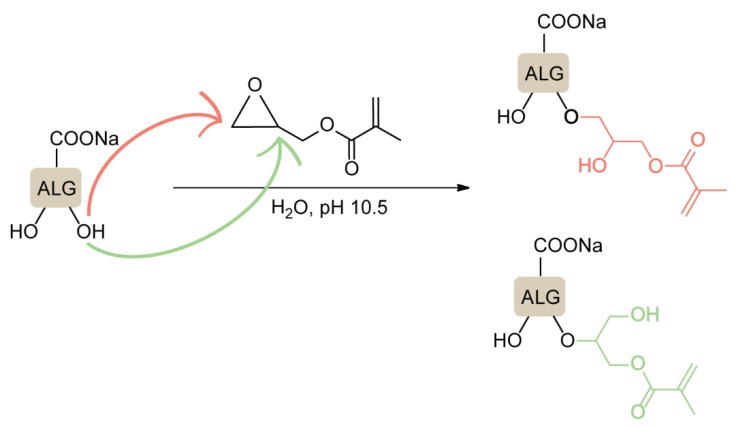
Epoxide ring-opening reactions of sodium alginate with glycidyl-methacrylate (GMA). Adapted from [59].

Additionally, ether formation through an epoxide ring-opening mechanism was used to prepare covalently cross-linkable alg derivatives with high absorption capacities for water treatment applications [59]. Na-alg was treated with glycidyl-methacrylate (GMA) at pH 10.5 in aqueous media, leading to a nucleophilic attack on both carbon atoms of the epoxide, while this type of reaction under basic conditions is expected to favor the attack at the less hindered position (Figure 6).

GMA-grafted alg derivatives were further covalently cross-linked with vinylated SiO_2_ MS to produce superabsorbent hybrid hydrogels that showed excellent removal capacity for water samples contaminated with methylene blue [59].

### 4.5. Synthesis of Graft Copolymers

**Figure 7:**
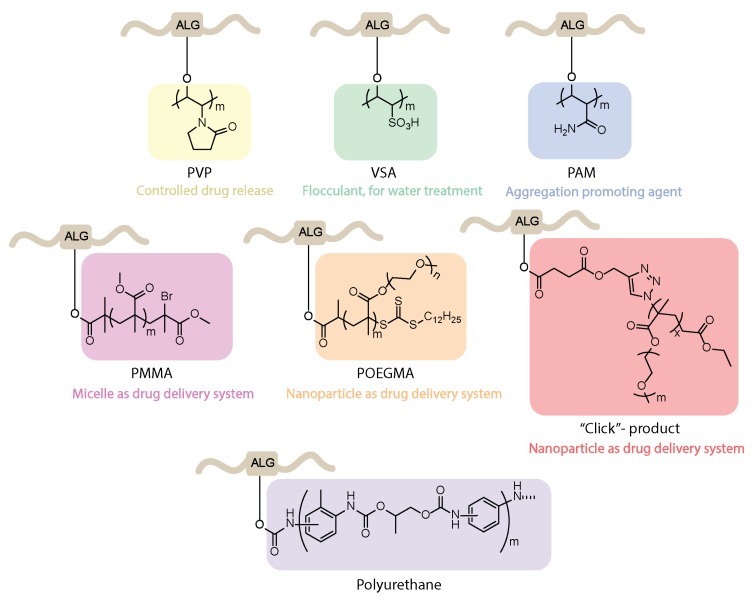
Examples of alginate graft copolymers and their applications.

Na-alg was successfully modified into different types of copolymers, taking advantage of the reactivity of its hydroxyl moieties to undergo graft polymerization in the presence of vinyl monomers and radical initiators (Figure 7). Azobisisobutyronitrile (AIBN) initiated *N*-vinyl-2-pyrrolidone (*N*-VP) grafting on Na-alg, followed by cross-linking with glutaraldehyde under acidic conditions, which led to the formation of Na-alg-*g*-PVP beads for the entrapment and subsequent release of the anti-inflammatory drug indomethacin [60]. Higher efficiency for both the encapsulation and the cumulative release of the drug was observed in comparison with pure Ca-alg cross-linked beads. The use of alg-based graft copolymers was also investigated in the context of flocculants production for water treatment. Free radical grafting of Na-alg with vinyl sulfonic acid (VSA) in the presence of the thiourea/peroxydiphosphate system [61] or with polyacrylamide (PAM) under microwave irradiation [62] provided aggregation-promoting agents with biodegradable and eco-friendly properties. 

Other methods of alg copolymer syntheses include single electron transfer living radical polymerization (SET-LRP) in the presence of a catalytic amount of Cu(0) or reversible addition-fragmentation chain transfer (RAFT) polymerization. TBA-alg was first esterified with bromoisobutyric acid and subsequently treated with polymethyl-metacrylate (PMMA) under SET-LRP conditions to produce self-aggregating micelles as potential smart drug delivery devices [63] that are suitable for post-functionalization on the free alg carboxylic groups. RAFT polymerization was used to produce copolymers of alg and poly(oligo ethylene glycol methacrylate) (POEGMA), which, in the presence of Ca^2+^ ions, formed nanoparticle-based encapsulation materials for 4-n-butylresorcinol [64]. An alternative route to alg-POEGMA copolymers made use of the azide to alkyne click-reaction, followed by nanoparticles self-assembly in the presence of Ca^2+^ ions, providing efficient encapsulation matrices for the chemotherapy drugs doxorubicin and paclitaxel [65]. Finally, alg hydroxyl functionalities were demonstrated to be suitable to generate grafted alg-*g*-polyurethanes by the polyaddition of isocyanates [66]. The reaction between the NCO terminated urethane prepolymer and TBA-alg was completed in organic solvent (DMF/DMSO) without catalyst. The methodology was applied to the extension of both ionic and non-ionic polyurethane polymers with TBA-alg.

### 4.6. Oxidation of Alginates

**Figure 8:**
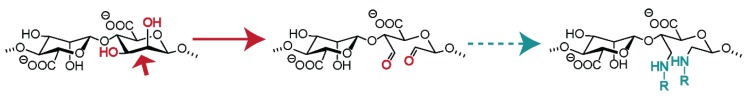
Oxidation of alginate and further transformation.

The oxidation of alg leads to the opening of the polysaccharide backbone rings between the vicinal diols, resulting in a more flexible polymer backbone and two highly reactive aldehyde groups. While oxidized alg derivatives have been extensively studied, further functionalization of the resulting carbonyl groups through reductive amination was frequently reported to achieve additional chemical modification of the polymer backbone (Figure 8).

#### 4.6.1. Oxidation without Further Modification

While the oxidation of alg has been studied since the 1970s [67,68,69,70,71], the real scope of this process emerged with the development of alg derivatives for tissue engineering applications, requiring finely tuned degradability. The most common reagent used for alg oxidation is sodium periodate (NaIO_4_), under aqueous, dark conditions. The degree of oxidation is limited by intramolecular hemiacetal formation between the so-formed aldehydes and nearby hydroxyl groups, thus preventing part of the alcohol functionalities to be further oxidized [67,72,73]. Despite the conservation of alg carboxylate groups during the process, ionic gelation is hindered above 10% oxidation of the polysaccharide backbone, with dependency on the initial alg molar mass and G/M ratio [72,74]. The oxidation of alg was found to go along with remarkable decrease of the viscosity of the resulting polymer solution, which could be attributed to two mechanisms. A fast process was found to involve the scission of atypical and infrequent monomer units of the alg backbone, leading to cleavage of the polysaccharide chain [70]. A slower reaction involving low concentrations of periodate-induced free hydroxyl radicals was also highlighted. Performing the reaction in ethanol/water dispersion instead of in pure water led to a reduction of the average molar mass of the resulting oxidized polymer, which was attributed to the contribution of hydroxyethyl radicals generated from ethanol [73]. Alternatively, the oxidation of alg was also performed using potassium permanganate (KMnO_4_) in the presence of H_2_SO_4_ as a catalyst [75]. Circular dichroism measurements gave evidence for the almost complete conversion of G units into dialdehydes, while the M units were susceptible to the cleavage of glycosidic bonds. 

For tissue engineering applications, the degradation rate of polymeric biomaterials should be precisely controlled and adjustable to the specific requirements of the intended application. The partial oxidation of alg (degree of oxidation of 1%) results in open chain derivatives that are susceptible to hydrolytic degradation [76]. Oxidized alg showed reduced gel stiffness and mechanical properties, with significant reduction of the ultimate stress and ultimate strain values. In addition, it was demonstrated that the degradation rate could be controlled without compromising gel stiffness simply by specific mixing of high and low molar mass oxidized alg derivatives, i.e., by adjusting the molar mass distribution of the alg derivatives [77].

#### 4.6.2. Chemical Modification of Oxidized Alginates

The use of oxidized alg derivatives for subsequent functionalization takes advantage of the high reactivity of the aldehyde moieties in comparison with the hydroxyl groups of native alg chains. 

**Figure 9:**
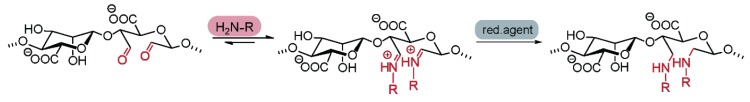
General procedure for reductive amination on oxidized alginates.

The most extensively reported reaction on oxidized alg–dialdehyde derivatives is the reductive amination (Figure 9). An overview of the types of functionalization introduced on the alg backbone by this method is presented in Table 2.

The common procedure involves imine formation, followed by reduction to the corresponding amine without isolation of the iminium intermediate. In order to avoid the reduction of non-reacted aldehydes, sodium cyanoborohydride (NaBH_3_CN) is the most common reagent for the reduction of the imine intermediates [78,79,80,81,82]. Following reductive amination on oxidized alg, post-functionalization of the resulting secondary amines was used to produce graft copolymers with PEG derivatives, resulting in promising candidates for microencapsulation applications [81]. Alternative reagents such as picoline-borane (pic-BH_3_) [83] were also reported as efficient and mild alg-imine reducing agents. Further reduction of the aldehyde moieties was achieved in the presence of ammonia-borane (BH_3_NH_3_) to produce degradable alg derivatives. This strategy was applied to the preparation of click cross-linked alg hydrogels for tissue engineering applications [84].

Numerous alg derivatives resulting from reductive amination were developed for drug delivery purposes. The conjugation of amino-thiophenol [85], cysteine [82], or cysteamine [86] to oxidized alg led to self-assembled nanospheres, cross-linked through disulfide bridge formation, which are sensitive to redox potential variations for controlled release of the entrapped drugs. Imine-functionalized alg-derived self-assembled nanoparticles were obtained by the conjugation of oxidized alg to doxorubicin and an additional encapsulation of curcumin for the co-delivery of both drugs to tumor cells, which was triggered by the pH variation in the tumoral environment [87]. Further applications of alg derivatives obtained by reductive amination with thiosemicarbazide [88] and cysteine [89] include the production of sorption materials for heavy metal removal from aqueous solutions. While the coordination with metal ions was shown to mainly involve the carboxylic groups of the functionalized polymers, the chemical modification with thiol-containing units was the key to the sorption properties, which was probably due to their role as cross-linkers between the polymeric chains [88].

Imine formation on oxidized alg derivatives was also applied to engineer alg/chitosan or alg/hyaluronic acid hydrogels for protein delivery [90] and cardiac tissue engineering [91]. 

**Figure 10:**
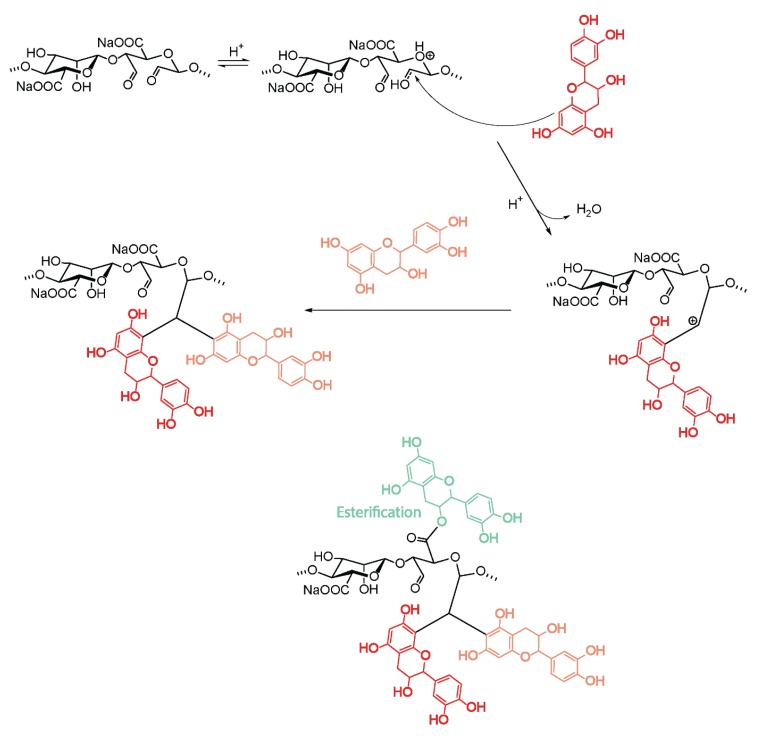
Conjugation of catechin to oxidized alginates.

Not only reductive amination was reported for the functionalization of oxidized alg. Oliver et al. demonstrated that the conjugation of the antioxidant catechin to alg-aldehyde under acidic conditions led to up to 22% functionalization with catechin, involving both nucleophilic addition to the carbonyl group and post-esterification of the carboxylate functionality (Figure 10) [92].

The resulting alg–catechin conjugates showed improved free-radical scavenging activity compared with non-functionalized Na-alg.

#### 4.6.3. Cross-Linked Hydrogels from Oxidized Alginates

**Figure 11:**
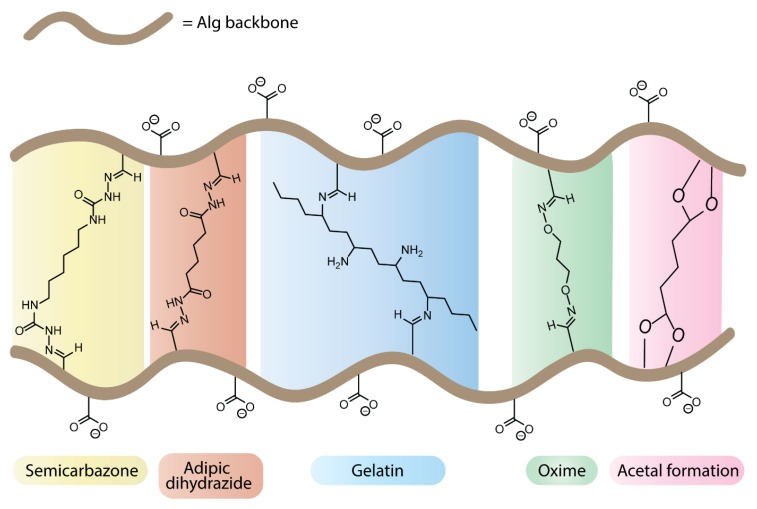
Cross-linked hydrogels from unmodified and oxidized alginates.

The oxidation of alg not only leads to polymers with biodegradable properties, but the resulting aldehyde functionalities can be used to produce cross-linked systems by bis–covalent conjugation with external cross-linkers (Figure 11). The choice of the type of chemical cross-linking can bring an additional level of control in tuning hydrogel degradability for tissue engineering applications or introduce extracellular matrix-like properties to alg-based materials.

The most common cross-linking strategy relies on the formation of bis-imine derivatives on oxidized alg. In the presence of adipic dihydrazide, low molar mass poly(aldehyde guluronate) segments were cross-linked through the formation of acyl hydrazone bonds, which are susceptible to hydrolytic cleavage in aqueous conditions [93]. The combination of chemical cross-linking and ionotropic gelation in the presence of Ca^2+^ ions provided an additional level of control over the mechanical properties of the resulting hybrid gels. While the degradation rate of these hydrogels is generally correlated to their cross-linking density, a detailed study of their degradation process under physiological conditions (37 °C, pH 7.4) revealed that hydrogels with a high content of hanging single-end molecules showed a retarded degradation process even at low cross-linking density, highlighting the opportunity for decoupled degradation rates and mechanical properties [94]. The cross-linking of alg-dialdehyde derivatives with gelatin [95] was reported to improve the cell–material interactions of alg-based hydrogels. The process involves imine formation with the ε-amino groups of lysine and hydroxylysine gelatin residues, at physiological pH [96] or in the presence of borax [97]. Depending on the alg to gelatin ratio and on the starting aldehyde content, a broad range of cross-linking densities was reported, as assessed by the degree of swelling and calculated from the Flory–Rhener equation [98,99]. The resulting hydrogels found applications for drug delivery and cell microencapsulation. In vitro cell interaction studies with human dermal fibroblasts in gelatin-cross-linked alg films showed high viability as well as enhanced cell attachment, spreading, and proliferation compared to non-modified alg material [100]. Investigation of the morphology and viability of MG-63 human osteosarcoma microencapsulated in gelatin-cross-linked alg hydrogels revealed an improved proliferation, migration, and formation of cellular network, as well as increased metabolic activity, in comparison with pure alg microencapsulated cells [101]. Further studies highlighted the faster degradation of gelatin–cross-linked alg hydrogels in comparison with pure alg hydrogels and gelatin–alg blends [102,103]. These cross-linked systems were also used for the development of 3D tumor models to study the behaviour of colon cancer cells [104]. Finally, cross-linked hydrogels with high gelatin content promoted the formation of filopodial protusions during the osteogenic differentiation of microencapsulated human adipose-derived stem cells [105]. Other imine-based cross-linking strategies include the formation of oxime and semi-carbazone functionalities, which showed promising properties for bioprinting applications [106]. Alternatively, unmodified alg was cross-linked by bis-acetalization with glutaraldehyde, under acidic conditions. The resulting hydrogels showed pH responsive swelling/contraction properties [107,108].

### 4.7. Other Functionalization Methods

In addition to the functionalization methods presented in the previous sections, a number of specific strategies were developed for the derivatization of alg hydroxyl groups.

A guest-responsive polymeric material was produced by the conjugation of 6-amino-α-cyclodextrin (CD) to Na-alg hydroxyl groups activated in the form of cyanate esters [109]. The degree of grafting was found to be dependent on the ratio of cyclodextrin to Na-alg, as well as on the reaction temperature, reaching a maximum of DG = 1.58. In the presence of Ca^2+^ ions, the CD-conjugated alg formed stable hydrogel MS, which were investigated as encapsulation material for the bacteria Sphingomonas cloacae in the context of the degradation of nonylphenol, which is an organic pollutant found in aquatic environment as a result of industrial surfactant biodegradation. The degrading ability of Sphingomonas cloacae toward nonylphenol as well as bacterial cell adhesion within the hydrogel matrix were superior to pure alg-MS in CD-conjugated alg-MS [110]. 

**Figure 12:**
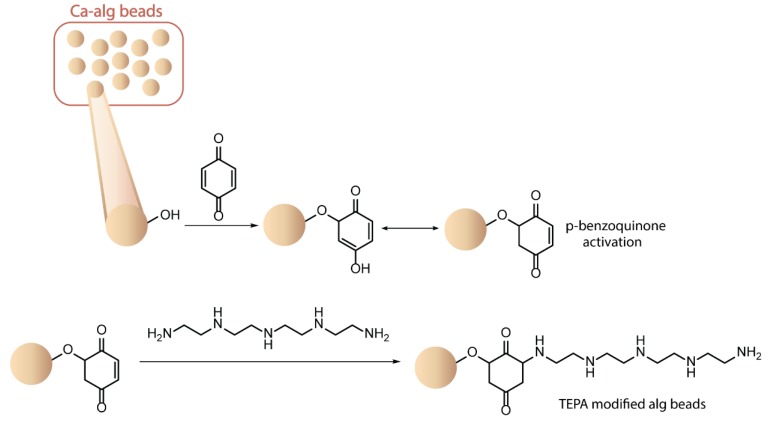
Modification of calcium alginate microspheres by activation of surface hydroxyl groups. Adapted from [112].

The alg hydroxyl groups were also functionalized with reactive groups increasing the sorption capacity of the native polysaccharide toward metal ions found in industrial effluents. Direct conjugation with urea and biuret significantly enhanced the ion-exchange capacity of the resulting MS toward toxic heavy metal ions such as Cd(II) and Pb(II), in comparison with pure alg beads [111]. Alternatively, Ca-alg MS were post-functionalized by the activation of surface hydroxyl groups in the presence of *p*-benzoquinone, followed by reaction with tetraethylenepentamine (Figure 12) [112]. The addition of amino groups covalently conjugated at the surface of alg beads, as confirmed by FT-IR studies, improved the removal capacity of the native hydrogel toward Cr(IV) ions in aqueous solutions. Interestingly, the amino-functionalized Ca-alg MS were found to be reusable, still possessing more than 80% of their initial adsorption capacity after five consecutive treatment cycles.

**Figure 13:**
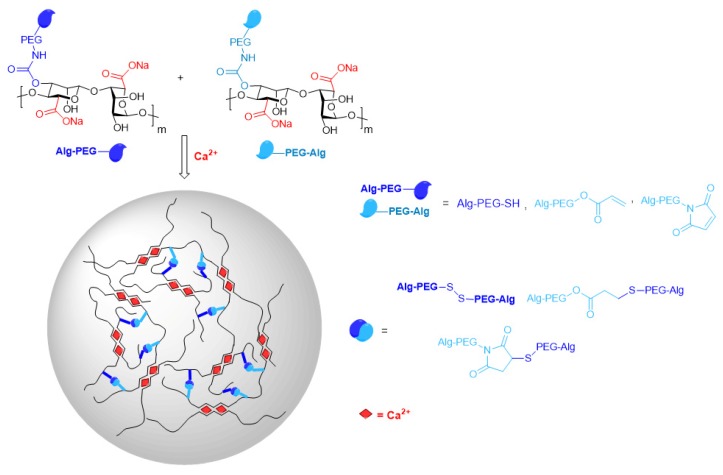
Dual ionic-covalent alginate–poly(ethylene glycol) (PEG) spherical hydrogels.

In the field of cell transplantation applications, the chemical modification of alg hydroxyl groups was investigated to improve the in vivo mechanical and chemical durability and to reduce the immunogenicity of the transplanted materials. A robust functionalization strategy was recently reported for the direct activation of alg hydroxyl moieties in the presence of 1,1-carbonyldiimidazole, followed by conjugation with cross-reactive PEG derivatives through a carbamate linkage [113]. The resulting alg–PEG polymers are simply mixed before extrusion into a Ca^2+^ containing gelation bath to produce hybrid MS assembled through simultaneously occurring fast ionotropic gelation and slower covalent cross-linking, i.e., slow thiol to thiol or thiol to carbon electrophile (Michael addition) bond formation, without the need for an additional chemical cross-linker (Figure 13) [114]. Optimization of the reaction conditions allowed finetuning of the molar mass of the alg–PEG polymers and precise control of the degree of grafting. The latter was deduced from ^1^H-NMR spectra by applying a deconvolution technique with Lorentzian functions to overcome the complexity of interpretation of overlapping signals. In comparison with pure Ca-alg MS, the dual ionic-covalent MS demonstrated higher mechanical resistance to uniaxial compression to 90% of their initial volume, improved shape recovery performance upon 10 repetitive compressions, and increased durability in aqueous solution containing non-gelling monovalent cations. The usability of the alg–PEG MS for cell encapsulation was validated on mouse insulinoma MIN6 cells and human hepatocellular carcinoma Huh7 cells. The cells maintained viabilities higher than 85% and functional secretion took place up to seven days after encapsulation [113,114].

PEGylation of alg hydroxyl groups aiming at the covalent conjugation of the anti-inflammatory drug ketoprofen to the hydrogel matrix of alg-based MS was also reported. For an in vivo comparison, ketoprofen-modified MS and pure alg beads, both containing encapsulated insulin producing MIN6 cells, were transplanted under the kidney capsule of immune-competent mice. The sustained hydrolytic release of ketoprofen from the modified MS led to a significant reduction of pericapsular fibrotic overgrowth [115]. The nature of the covalent bond between the drug and the PEG fragment (ester, amide) governed the release kinetics, thus potentially providing an efficient strategy for the mitigation of adverse fibrotic response after the transplantation of encapsulated endocrine cells.

## 5. Outlook

**Figure 14:**
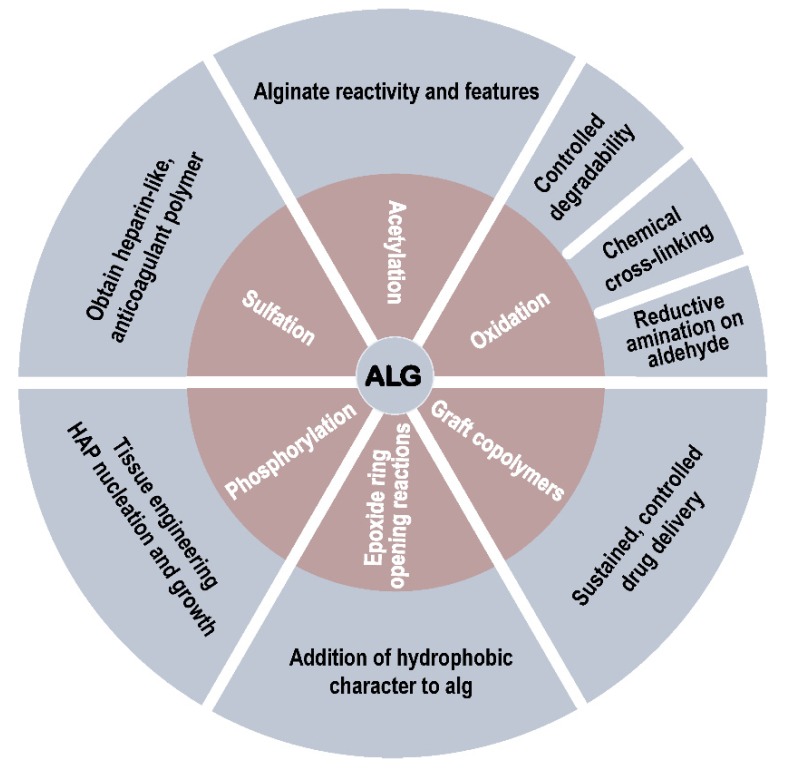
Functionalization of sodium alginate by reactions on the hydroxyl groups: an overview.

The presence of hydroxyl and carboxyl moieties on the backbone chain makes the natural anionic polyelectrolyte alg amenable to a large variety of chemical modifications and functionalizations aiming at the modulation of the physical, chemical, and biological properties. In order to maintain the full capacity of alg derivatives to spontaneously form hydrogels in the presence of divalent cations, the more promising functionalization strategies preferably focus on the selective modification of the hydroxyl moieties (Figure 14).

Despite the remarkable potential of alg derivatives for various applications, in particular in the biomedical domain, increasing the number of translations to the clinics remains a challenge. The scalability and reproducibility of the functionalization pathways, as well as reproducibility of the physicochemical characteristics of modified alg derivatives, need further improvement for meeting the regulatory requirements as prerequisites for commercial pharmaceutical and biomedical use.

The intention of this review is to provide a compact overview on the state of the art of Na-alg modification and functionalization without restriction of the polyelectrolyte properties in order to support ongoing and future research and development in this field.

It can be expected that ongoing and future research in the above addressed fields will profit not only from novel alg-based derivatives, but also from progress in analytical, characterization, and separation/purification methods and from novel manufacturing and processing technologies. On the one hand, the performance of analytical, characterization, and separation/purification methods is a key to more comprehensive regulatory compliance. On the other hand, novel computer design techniques and IT-supported technologies to transfer the alg derivatives into well-defined optimal shapes and macro structures are expected to accelerate their practical use. 

Examples of advanced manufacturing techniques include microfluidics and 3D or 4D bioprinting. Alg-based materials are prospective bioinks for bioprinting, where one of the biggest challenges is finding a good compromise between printability (polymer concentration) and cytocompatibility [116]. Chemical modification of alg materials into dynamic hydrogels [117], stimuli-responsive materials [118], or functionalization with drugs, growth factors, or biological clues could lead to the development of more sophisticated biologically functional 3D constructs. Due to the wide variety of the properties of alg derivatives, in particular those equipped with biological activity, even integration into organoids or whole printed artificial organs could be imagined [119], or fabrication of personalized and vascularized biomedical devices, implants, and grafts [120]. 

Apart from the field of tissue engineering and regenerative medicine, the pharmaceutical industry could also profit from these advances of bioprinting. Preclinical in vitro drug screening on mini-tissue, organ-on-a-chip, or tissue/organ constructs could accelerate the screening of potential new drugs [121].

Given the remarkable recent advances in synthesis strategies, analytics, characterization, and technology, it may be concluded that in particular, interdisciplinary and multidisciplinary approaches are the best drivers for transferring and implementing research and development results into commercial products, demanding pharmaceutical and medical applications, including novel clinical therapies.

## Figures and Tables

**Table 1:** Sulfation methods for alg hydroxyl groups derivatization. TBA: tetrabutylammonium.

Starting Polymer	Sulfation Agent	Application	Ref.
Na-alg	ClSO_3_H/formamide	Anticoagulant heparin analogue	[43]
Poly-G-alg	ClSO_3_H/formamide	Anticoagulant heparin analogue and anti-inflammatory compound	[44]
TBA-alg	DCC, H_2_SO_4_/DMF	Heparin-binding proteins release	[50]
Na-alg	N(SO_3_Na)_3_/H_2_O	Anticoagulant heparin analogue	[49]
Na-alg, various G:M ratios	ClSO_3_H/formamide	Evaluation of heparin-like properties of sulfated-alg	[45]
TBA-alg	SO_3_/pyridine	Chondrocyte encapsulation	[51]
Na-alg	ClSO_3_H/formamide	Chondrocyte encapsulation	[46]
Na-alg	ClSO_3_H/formamide	Nanofiber electrospinning	[47]
Alginic acid, Poly-M-alg and Poly-G-alg	ClSO_3_H/formamide	Structure-activity relationship of propylene glycol alginate sodium sulfate (PSS) derivatives	[48]

**Table 2:** Functionalization of oxidized alginates by reductive amination.

[Ox] Degree	R-NH_2_	Reducing Agent	Application	Ref.
20%	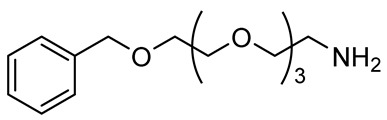	NaBH_3_CN	Amphiphilic alginate	[78]
40%	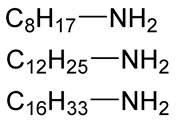	NaBH_3_CN	Hydrophobically modified alginate, surfactant, removal of heavy metals	[79]
30%	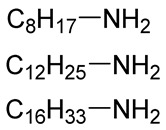	NaBH_3_CN	Hydrophobically modified alginate, polymeric surfactant	[80]
20%	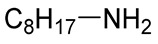 (Further modified with PEG)	NaBH_3_CN	High biocompatibility material	[81]
44%	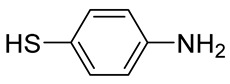	NaBH_4_	Drug delivery	[85]
n.d.	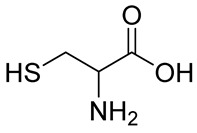	NaBH_3_CN	Drug delivery	[82]
8%	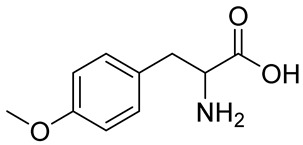 Peptide sequences: GRGDYP, GRGDSP, KHIFSDDSSE	pic-BH_3_	Material with cell attachment sites, induction of cell interaction	[83]
48%	Doxorubicin	-	Self-assembly pH sensitive nanoparticle for drug delivery	[87]
n.d.	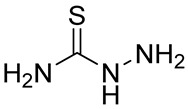	NaBH_4_	Heavy metal removal from water	[88]
30%	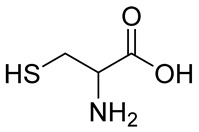	NaBH_4_	Heavy metal removal from water	[89]
3–11%	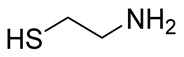	NaBH_4_	Drug delivery	[86]
